# Balancing the Positives and Negatives: A Bibliometric Analysis of Positive and Negative Study Publication Patterns in High-Impact General Medical Journals

**DOI:** 10.7759/cureus.108720

**Published:** 2026-05-12

**Authors:** Benjamin S Vipler, Cameron Arnet, Angela Keniston, Matthew Mardo, Christopher J King

**Affiliations:** 1 Department of Medicine, University of Colorado School of Medicine, Aurora, USA

**Keywords:** bibliometric analysis, general internal medicine, negative studies, publication bias, randomized controlled trial

## Abstract

Despite calls to publish negative studies in prominent medical journals, greater submission and acceptance of positive results remains an issue. We aimed to quantify the degree to which high-impact general medical journals publish negative study results. We searched MEDLINE/PubMed for all randomized controlled trials published in five high-impact general medicine journals: Annals of Internal Medicine, the British Medical Journal (BMJ), the Journal of the American Medical Association (JAMA), the Lancet, and the New England Journal of Medicine (NEJM). Our search spanned a 10-year period from 2014 to 2023, which included data from before and after the emergence of the COVID-19 global pandemic. We performed single-author data extraction via abstract review to determine study positivity, defined as statistical significance for the primary outcome, flagging abstracts for secondary review if positivity was not clear. Two authors reviewed all flagged abstracts. We calculated the proportion of negative studies (i.e., not meeting statistical significance for the primary outcome) overall, by journal, and by publication year. We used logistic regression to model the odds of a study reporting a negative result by journal and year. Our search yielded 3722 individual citations, with screening resulting in 3600 randomized controlled trials for review, with 31% of studies reporting negative results. The proportion of negative studies varied, ranging from 22% in the Lancet to 51% in BMJ and JAMA. The proportion of negative studies remained consistent over time. High-impact general medical journals vary widely in the percentage of negative studies that they publish but did not change over time, even during and after a global pandemic. Further study is needed to determine factors influencing this phenomenon and what can be done to minimize publication bias.

## Introduction and background

Despite multiple calls encouraging the publication of studies with negative results [1-3], publication bias, that is, the propensity for authors to submit and/or journals to accept manuscripts with positive, statistically significant results, remains an issue in academic publishing [4]. Only 53% of completed trials are ever published, and those with statistically positive results are 2.69 times more likely to be successfully published [5]. It is unclear if high-impact medical journals have changed publication practices over time to mitigate the risk of publication bias. 

The effects of publication bias have been documented extensively. Having an evidence base that is skewed towards positive studies, defined as those with statistically significant findings for the primary outcome, can lead clinicians to overestimate treatment effects [6]. Many negative trials, defined as those that do not show statistical significance for the primary outcome, provide data that definitively answer a scientific question. Approximately 70% of non-significant results from trials published in leading journals had likelihood ratios exceeding 10 in favor of the null hypothesis, with 50% exceeding 100 and 30% exceeding 1000 [7]. In order to reduce the effect of publication bias, formal literature reviews have methodologies that allow for modeling to recognize when publication bias exists around a given topic [8]. In the absence of formal literature reviews, it is difficult for researchers and clinicians to quantify publication bias related to each unique clinical or scientific query that arises.

Publication of a mixture of positive and negative studies provides a more complete scientific picture. During the COVID-19 pandemic, an opportunity to publish both positive and negative studies presented itself to the academic community. With the novel challenge of a new viral vector, scientific study rapidly evolved, and both positive and negative studies contributed to our understanding of transmission and how to treat patients. This time period offered a potential catalyst to change, or accelerate the change in, the publication patterns of medical journals. In order to better characterize the frequency of publication of statistically negative randomized controlled trials (RCTs), both before and after the emergence of COVID-19, we sought to quantify the degree to which high-impact medical journals publish statistically positive versus negative results of RCTs through a bibliometric analysis. 

## Review

Methods

Data Sources, Search String, and Study Selection

We searched the National Library of Medicine database via MEDLINE/PubMed for all RCTs published in five high-impact general medicine journals (Annals of Internal Medicine, the British Medical Journal (BMJ), the Journal of the American Medical Association (JAMA), the Lancet, and the New England Journal of Medicine (NEJM)). Our review spanned a 10-year period (1/1/2014-12/31/2023) with dates chosen to allow for full calendar years given data extraction began in 2024. We selected these five journals based on previous studies examining publishing practices of high-impact medical journals [9,10]. Our search string was as follows: ("Annals of internal medicine"[Journal] OR "The New England journal of medicine"[Journal] OR "lancet london england"[Journal] OR "JAMA"[Journal] OR "bmj clinical research ed"[Journal]) AND (("2014/01/01"[Date - Publication]: "2023/12/31"[Date - Publication])). We filtered results by RCTs after running this search string and reviewed all results returned. Abstracts for studies not meeting the criteria of an RCT were excluded, and the categories and number of studies in each excluded category are shown in a figure below. 

Determination of Study Positivity or Negativity

Journal abstracts were reviewed by one of four authors (BV, CA, MM, or CK) to determine whether individual studies were positive or negative. Studies that were not clearly positive or negative were flagged for secondary review by two co-authors (BV, CK) who reached consensus on the categorization (positive or negative) of each abstract. After secondary dual author abstract review of ambiguous studies, all disagreements were resolved. Studies were defined as positive if there was a statistically significant difference in one or more of the primary outcomes in unadjusted statistical analyses. We defaulted to a p-value of ≤0.05 as statistically significant unless otherwise specified in the individual research study. If clinical and statistical significance were noted in the manuscript, we defaulted to statistical significance unless there was a prespecified margin of efficacy. Studies that were inconclusive or underpowered (e.g., enrollment difficulties) were coded as negative. If an abstract defined a hypothesis and achieved a statistically significant result that was opposite to that hypothesis (e.g., a hypothesis that testosterone decreases plaque size but the study showed testosterone increased plaque size), the study was coded as negative. Studies without primary outcomes reported or defined were excluded. 

For non-inferiority trials, a result of non-inferiority was coded as positive as this was the goal of the study. Similarly, in comparative effectiveness trials, if the researchers' hypothesis was that the intervention and control are equal and the study showed equality, the study was coded as positive. If there was no clear hypothesis for equivalence, then a result that was not statistically significant was coded as positive (similar to a non-inferiority study). Vaccine and drug trials were coded as follows: positive if safe and effective; negative if unsafe, regardless of effectiveness; negative if ineffective, regardless of safety; and positive if safe, but efficacy was not reported. Only human data was reviewed to determine positivity. 

Statistical Analysis

We calculated the proportion of negative studies overall, by journal, and by publication year. We generated a bar chart illustrating the proportion of negative articles by journal and a line graph showing annual trends in the proportion of negative studies. Logistic regression was used to model the odds of a study reporting a negative result by journal and year. Standard logistic regression assumptions were considered, including independence of observations and correct specification of the logit. The calendar year was centered at 2019 to make the model intercept interpretable. We included an interaction term between journal and year to assess whether temporal trends differed across journals. Year was modeled as a continuous variable to capture linear trends, and predicted probabilities were derived to visualize differences in publication patterns by journal over time. Model goodness of fit was assessed using the deviance and Pearson chi‑squared goodness‑of‑fit statistics and the Hosmer-Lemeshow chi‑squared test. There was no evidence of lack of fit based on deviance, Pearson, or Hosmer-Lemeshow goodness‑of‑fit tests (all p>0.05). Because a single prespecified logistic regression model was fit, no adjustment for multiple testing was applied. Model results are reported in a table below as coefficient estimates (log‑odds), standard errors, odds ratios, 95% confidence intervals, and corresponding two‑sided p‑values. All analyses were conducted in SAS Enterprise Guide 8.3 (SAS Institute, Inc., Cary, North Carolina, United States).

Results

**Figure 1:**
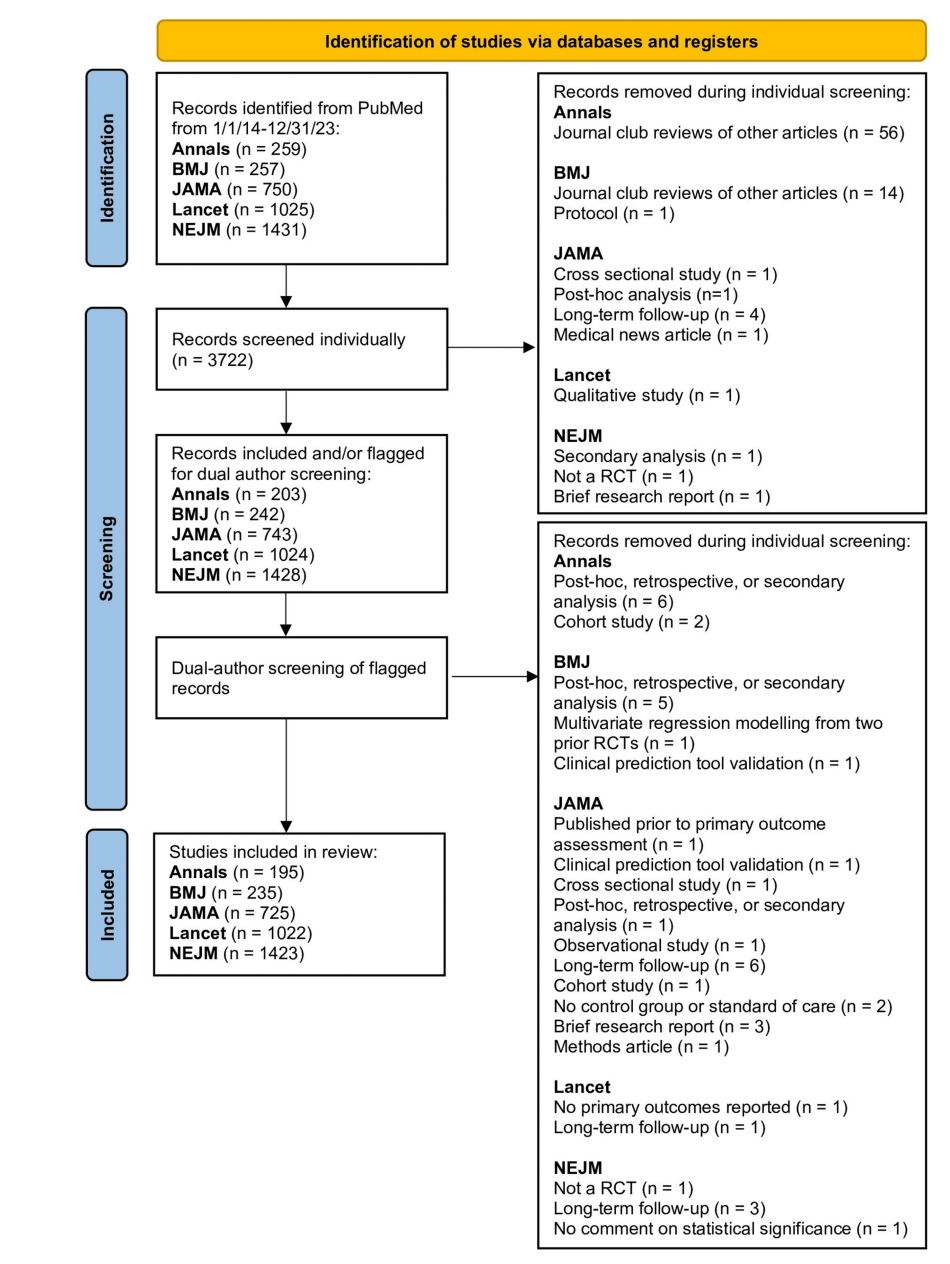
Identification, screening, exclusion, and inclusion of RCTs Annals: Annals of Internal Medicine; BMJ: British Medical Journal; JAMA: Journal of the American Medical Association; NEJM: New England Journal of Medicine; RCT: randomized controlled trial

Our search criteria for RCTs across the five target medical journals yielded 3722 individual citations. Through screening and exclusion (Figure 1), we ultimately included 3600 studies for review.

**Figure 2:**
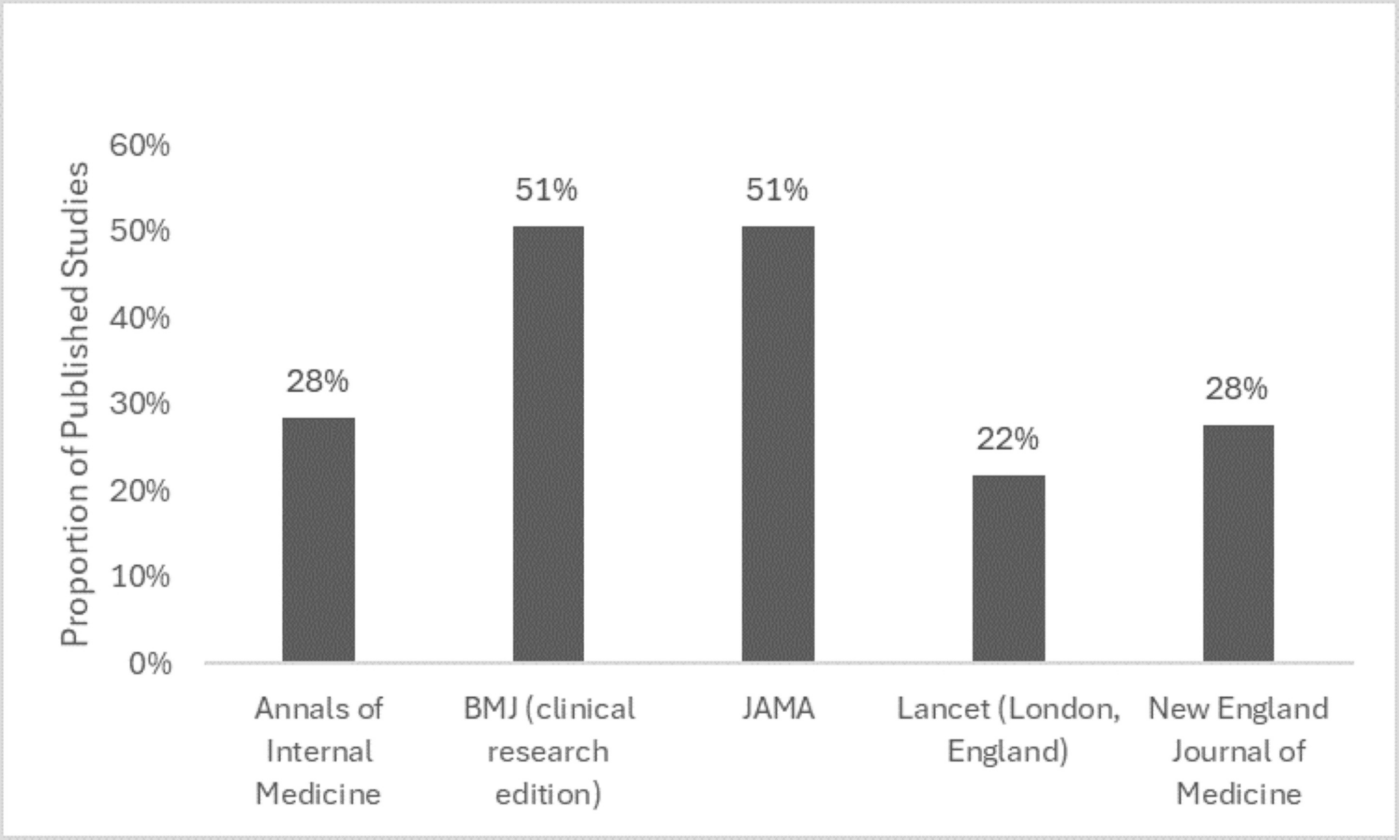
Proportion of negative studies published by journal (2014-2023) BMJ: British Medical Journal; JAMA: Journal of the American Medical Association

For the studies reviewed, 31% reported negative results. Across the five journals, the proportion of negative studies varied, ranging from 22% in the Lancet to 51% in BMJ and JAMA (Figure 2).

**Figure 3:**
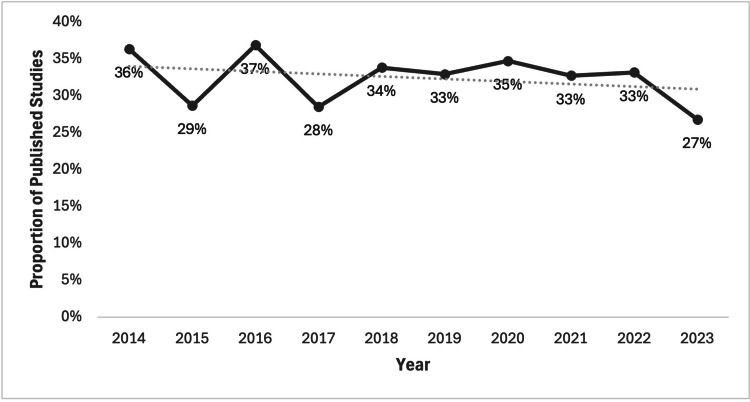
Negative study publication percentage (2014-2023) Percentage of trials published showing negative results across all five target journals.

Over time, the total proportion of negative studies from all five journals remained consistent (Figure 3).

In a logistic regression model including an interaction between journal and year, with NEJM as the reference category, trends in the likelihood of publishing negative studies differed across journals over time (p=0.0236). Compared to NEJM, JAMA demonstrated a significantly greater increase over time in the odds of publishing negative studies (interaction OR= 1.10; 95% CI: 1.03-1.17; p=0.0063). Similarly, compared to NEJM, the Lancet demonstrated a significantly greater increase over time in the odds of publishing negative studies (interaction OR= 1.09; 95% CI: 1.02-1.16; p=0.0157). Annals and BMJ did not differ significantly from NEJM in their trends over time. Table 1 presents the results for each journal for the logistic regression model. For interpretability, year was centered at 2019 when calculating odds ratios and confidence intervals.

**Table 1:** Logistic regression modeling of the odds of a study reporting a negative result by journal and year Results of a logistic regression model which included an interaction term between journal and year, centered on 2019, to assess whether temporal trends differed across journals. Year was modeled as a continuous variable to capture linear trends, and predicted probabilities were derived to visualize differences in publication patterns by journal over time. Model fit statistics: deviance χ²(df=40)=49.4 (p=0.1462); Pearson χ²(df=40)=49.1 (p=0.1531); Hosmer-Lemeshow χ²(df=9)=2.2 (p=0.9869). N=3595. BMJ: British Medical Journal; JAMA: Journal of the American Medical Association

Parameter	Estimate (log‑odds, 95% CI)	Standard error	Odds ratio (95% CI)	P-value
Intercept	-0.9919 (-1.111, -0.8728)	0.0608	-	<0.0001
Journal				
Annals of Internal Medicine	0.0541 (-0.2845, 0.3926)	0.1727	1.06 (0.75, 1.48)	0.7543
BMJ (clinical research edition)	1.0165 (0.7337, 1.2994)	0.1443	2.76 (2.08, 3.67)	<0.0001
JAMA	1.0380 (0.8480, 1.2279)	0.0969	2.82 (2.34, 3.41)	<0.0001
Lancet (London, England)	-0.2605 (-0.4526, -0.0684)	0.0980	0.77 (0.64, 0.93)	0.0079
New England Journal of Medicine	Reference			
Year	-0.0601 (-0.1010, -0.0192)	0.0209	0.94 (0.90, 0.98)	0.0040
Interaction term				
Year * Annals of Internal Medicine	-0.0213 (-0.1346, 0.0919)	0.0578	0.98 (0.87, 1.10)	0.7119
Year * BMJ (clinical research edition)	0.0561 (-0.0375, 0.1497)	0.0478	1.06 (0.96, 1.16)	0.2404
Year * JAMA	0.0914 (0.0259, 0.1569)	0.0334	1.10 (1.03, 1.17)	0.0063
Year * Lancet (London, England)	0.0816 (0.0154, 0.1478)	0.0338	1.09 (1.02, 1.16)	0.0157
Year * New England Journal of Medicine	Reference			

**Figure 4:**
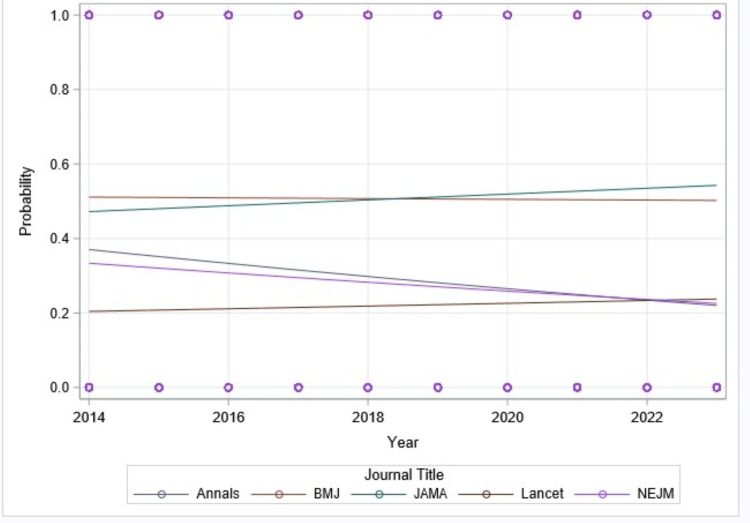
Predicted probabilities for published trial being negative Annals: Annals of Internal Medicine; BMJ: British Medical Journal; JAMA: Journal of the American Medical Association; NEJM: New England Journal of Medicine

Figure 4 illustrates these trends over time by journal.

Discussion

Each scientific domain has a different risk of publication bias [11], and to our knowledge, this is the most robust bibliometric analysis over a 10-year period of high-impact general medical journals, showing that there are differences in the publication rates of negative studies across journals. Over the period studied, certain journals published over twice the proportion of negative studies, highlighting the variability in publication practices among high-impact journals. Negative publication rates remained steady over time suggesting even the emergence of a global pandemic was not sufficient to change the publication practices of these high-impact medical journals. There are journals that are working to mitigate positive study publication bias though, which was seen with two journals having increased odds of publishing negative studies when compared to NEJM. These efforts should be encouraged by the academic community and the larger scientific community.

Our study has implications for clinicians, scientists, and journal editors. The stability of publication rates of negative results should give clinicians pause when considering whether our understanding of a particular research question is incomplete due to current publication practices. This same pause will hopefully prompt more formal literature reviews that can help identify if publication bias is likely present, as our study cannot answer this question. Scientists can utilize these results to determine which journals may be more likely to accept and publish negative results. It is worth noting that without changes to how negative study manuscripts are reviewed and accepted, these results may lead to the reinforcement of current practices as authors preferentially submit negative results to those journals that are more likely to publish their work, entrenching the current differences in publication practices. Ideally, editorial boards for all journals can use our results to begin exploring their own practices around the evaluation and publication of negative results, as surveys of journal editors have shown that positive studies are more likely to achieve publication than negative studies [12].

Our findings align with findings from other fields, underscoring that negative study results continue to have difficulty getting published in peer-reviewed journals [13], not just in general internal medicine journals. Negative studies, even when published, suffer from significant lag-time bias with publication occurring in 2.6 years versus two years for positive studies [5]. When investigators evaluate published data to understand the gaps in our current knowledge, the lack of opportunity and delay in publishing negative studies clouds the picture. Investigators may pursue studies that have already been completed by others, were negative, and therefore were not published. Because the negative findings were not disseminated, others may repeat these trials. This may lead to patients receiving ineffective or harmful care due to the repeated exploration of a hypothesis where data was not published due to a lack of statistical significance [14].

While our findings are important, our study has multiple limitations. We only reviewed five high-impact factor journals rather than all peer-reviewed general medical journals. Negative studies that were rejected from one or all of these journals may have been published in a different journal not included in our review. While we are able to draw conclusions about the proportion of negative and positive studies in this analysis, we cannot draw definitive conclusions about the presence of submission bias, editorial bias, or publication bias with this data set. Due to feasibility, we did not compare our database search results with clinical trial registries. Thus, we do not have information on the consequences of these studies. Our review strategy was also limited in that we only had a single-author review of abstracts with arbitration only if the primary reviewer highlighted the need. While there is the possibility that abstracts could be incorrectly categorized leading to misclassification bias, given the large number of abstracts reviewed, the overall percentages of published negative studies would have likely remained similar. We also did not calculate inter-rater reliability statistics between reviewers. Our study has several strengths including a robust and consecutive 10-year sample size that includes RCTs from a wide array of disciplines and spans time before and after the emergence of COVID-19. The large sample size allowed us to look at individual journal practices as well as trends by year.

## Conclusions

High-impact factor general medicine journals publish negative studies at widely different rates which were not impacted by the COVID-19 pandemic. This review highlights the need for continued improvement in the submission, review, and publication of negative RCTs. Whether driven by individual journals or via international committee recommendations, publication of negative studies must continue to improve the publication patterns of major medical journals. 
